# Patient-Reported Outcomes following Tibial Plateau Fractures: Mid- to Short-Term Implications for Knee Function and Activity Level

**DOI:** 10.3390/jcm13082327

**Published:** 2024-04-17

**Authors:** Claas Neidlein, Julius Watrinet, Robert Pätzold, Daniel P. Berthold, Wolf Christian Prall, Wolfgang Böcker, Boris Michael Holzapfel, Julian Fürmetz, Markus Bormann

**Affiliations:** 1 Department of Orthopedics and Trauma Surgery, University Hospital, LMU Munich, Musculoskeletal University Center Munich (MUM), Marchioninistraße 15, 81377 Munich, Germany; claas.neidlein@med.uni-muenchen.de (C.N.);; 2 Department of Trauma Surgery, Trauma Center Murnau, Professor-Küntscher-Str. 8, 82418 Murnau, Germany; 3 Devision of Knee, Hip, Shoulder and Elbow Surgery, Schoen Clinic Munich, Harlachinger Straße 51, 81547 Munich, Germany

**Keywords:** tibial plateau fracture, patient-reported outcome, return to sports, post-traumatic osteoarthritis

## Abstract

**Background**: Patients with complex proximal tibial plateau fractures (TPFs) tend to overestimate the prognosis of their injury, potentially due to factors such as a limited understanding, optimism, and the influence of the pain intensity. Understanding the reasons behind this misperception is crucial for healthcare providers to effectively communicate with patients and establish realistic expectations for treatment outcomes. The purpose of this study was to analyze the outcomes of TPFs, with a particular focus on patient-reported outcome measures concerning functional recovery, pain levels, and overall satisfaction with treatment. The authors aim to provide valuable insights into the realistic expectations and potential limitations that patients may encounter during their recovery journey. **Methods**: In this retrospective single-center study, all surgically treated TPFs between January 2014 and December 2019 with a minimum follow-up of 12 months were included. Several patient-reported outcome measures were obtained, including the International Knee documentation Committee Score (IKDC), Lyholm score, Tegner score, and visual analog scale (VAS) for pain. Fractures were classified according to Schatzker, and then subgrouped into simple (Schatzker I–III) and complex (Schatzker IV–VI) fractures. **Results**: A total of 54 patients (mean age 51.1 ± 11.9 years, 59.3% female) with a mean follow-up time of 3.9 years were included. Schatzker II fractures were present in 48% (*n* = 26) of the cases, with Schatzker III in 6% (*n* = 3), Schatzker IV fractures in 6% (*n* = 3), and Schatker VI fractures in 41% (*n* = 22) of the cases. All outcome scores showed a significant improvement between the first year after surgery and the last follow-up (mean: 3.9 years). Simple fractures showed significantly lower patient-reported outcomes when compared to the preinjury state; however, good to excellent results were observed. Patient-reported outcomes of complex fractures showed no significant changes in the study period with good to excellent results. When it comes to the Lysholm score, there were no significant differences in the outcome between simple and complex fractures. Furthermore, there was a return-to-sports rate of 100%, with high rates of changing sporting activity in 25% (simple fractures) and 45% in complex fractures. **Conclusions**: The data from this study showed that both simple and complex tibial plateau fractures show favorable outcomes at the midterm follow-up, and that injury severity does not correlate with worse results. While patients may tend to overestimate the recovery speed, this research highlights the importance of long-term follow-up, demonstrating a substantial improvement between one year post-surgery and the final evaluation. Return-to-sports rates were high, with adjustments needed for certain activities. However, patients should recognize the need to shift to lower-impact sports and the lengthy recovery process.

## 1. Introduction

Tibial plateau fractures (TPFs) are complex injuries that may greatly impact patients’ daily lives and functional abilities. An accurate assessment of the prognosis and a comprehensive comprehension of potential limitations during recovery are essential for both surgeons and patients. However, studies have shown that patients often overestimate the prognosis of their injuries, which leads to unrealistic expectations and potential dissatisfaction with treatment outcomes [1].

TPFs account for 1% of all fractures [2], but there has been a recently described increase in incidence, reaching up to 28.7 per 100,000 persons per year [3,4,5]. The majority of these fractures are treated surgically [4], and several surgical strategies have been described to achieve the optimal anatomical restoration of the joint plane and alignment of the axis. Fractures with a persistent articular surface depression greater than 10 mm [6], as well as infections and mal- or nonunion [7], are significant predictors for a poor outcome. Despite optimal surgical therapy, secondary osteoarthrosis is reported in 44% of the cases, leading to high complication rates [8]. In recent years, multiple surgery rates from different countries have been described in the literature, ranging from 37% (Belgium) to 92% in Denmark [4,9,10]. Unfortunately, there is a lack of information on non-surgical treatment and the importance of postoperative rehabilitation on TPFs.

The literature categorizes TPFs into high-/complex- and low-/simple-energy fractures [11,12]. Interestingly, there is no significant correlation between radiological and functional outcomes [13,14]. However, the majority of patients can achieve good to excellent functional results in the short and long term [13,15,16,17,18,19,20,21]. Nevertheless, the literature on patient-reported outcomes of TPFs is limited, and further research is necessary to fully understand the impact of these severe injuries on patients.

In view of the disparity between patients’ perceptions and actual outcomes, there is a pressing need for comprehensive research that analyzes the true patient-reported data regarding proximal tibial plateau fractures, particularly given the significant increase in TPFs [4,22]. By gaining a deeper understanding of the functional limitations and recovery trajectories associated with these fractures, surgeons can better inform patients about the expected outcomes and assist them in making well-informed decisions regarding their treatment options.

The purpose of this study was to analyze the outcomes of TPFs, with a particular focus on patient-reported outcome measures concerning functional recovery, pain levels, and overall satisfaction with treatment. The authors aim to provide valuable insights into the realistic expectations and potential limitations that patients may encounter during their recovery journey. This information can significantly enhance shared decision-making between surgeons and patients, ultimately leading to improved patient satisfaction and optimized outcomes. The hypothesis was that patient-reported outcomes following surgically treated TPFs are not solely dependent on fracture classification, and both simple and complex fractures will yield favorable outcomes, enabling patients to maintain an active lifestyle.

The study complies with the ethical principles of the Declaration of Helsinki and was approved by the local ethics commission (21-0559).

## 2. Materials and Methods

### 2.1. Patient Selection

In this retrospective single-center study, all surgically treated TPFs between January 2014 and December 2019 (*n* = 319) with a minimum follow-up of 12 months were included. Inclusion criteria comprised surgically treated tibial plateau fractures (TPFs) with a minimum follow-up duration of 12 months, intra-articular unilateral fractures, isolated fractures of the affected limb, preoperative radiographic and/or computed tomographic imaging, and a minimum age of 18 years. Criteria for exclusion were bilateral TPFs, not classified according to Schatzker (Moore 3), and patients who could not be contacted, were not available for follow-up (e.g., due to a change in address, language barrier, or lack of proficiency in German or English), or refused participation. The study complies with the ethical principles of the Declaration of Helsinki and was approved by the local ethics commission (21-0559).

Demographics were collected through chart review. Fractures were classified according to Schatzker [23], utilizing X-ray and/or computed tomography imaging, and subdivided into simple (Schatzker I–III) and complex (Schatzker IV–VI) fractures. The institutional research group, consisting of one head of department and one scientific assistant, performed the fracture classifications, and disagreements among the raters were resolved through discussion.

### 2.2. Surgical Treatment

#### 2.2.1. Osteosynthetic Treatment

Fifty-one patients (94.4%) were treated by open reduction and internal fixation (ORIF), while 13 (24.1%) received additional arthroscopic surgery. In 5.6% (*n* = 3) of the cases, screws were used for treatment. An anterolateral standard approach was employed in 63% (*n* = 34) of the cases, while an isolated posterior approach was used in 9.3% (*n* = 5) of the cases and an isolated medial approach was used in 13% (*n* = 7) of the cases. Additionally, 9.3% (*n* = 5) were treated with a combined approach.

In the overall cohort, 24% of the patients were treated non-surgically.

#### 2.2.2. Bone Grafting

Overall, a total of 22 patients were treated (40.7%) by additional bone grafting. The most common types were autograft (*n* = 9; 40.9%) and pure allograft (*n* = 6; 27.3%). A combination of allogenic and autologous grafts was used in 4.5% (*n* = 1) of the cases, and synthetic bone graft was also used in 27.3% (*n* = 6).

#### 2.2.3. Meniscal and Ligament Repair

In total, 20 patients (37.0%) received single-stage meniscus and/or ligament repair. Among these, 20.4% (*n* = 11) were treated for meniscal injuries, 13.0% (*n* = 7) for anterior cruciate ligament (ACL) injuries, 5.6% (*n* = 3) for lateral collateral ligament (LCL) injuries, 1.9% (*n* = 1) for posterior cruciate ligament (PCL) injuries, and 1.9% (*n* = 1) for medial collateral ligament (MCL) injuries.

### 2.3. Patient-Reported Outcomes

The International Knee Documentation Committee score (IKDC) [24], Lysholm score [25], visual analog scale for pain (VAS), and the Tegner score [25] were used to assess the patients. Surveys were conducted pre-injury and at a minimum of 1 year postoperatively. Return to sports (RTS) was evaluated using the Tegner score, where a score of 3 indicated engagement in sporting activities such as competitive and recreational sports (e.g., swimming, and walking in the forest). The range of motion (ROM) of both the injured and healthy knees was measured in degrees using a goniometer (medi GmbH & Co. KG, Medicusstraße 1, 95448 Bayreuth, Germany) for flexion and extension. Moreover, intraoperative, and postoperative complications were collected.

### 2.4. Statistical Analysis

Count and percentage were used to report categorical variables. Using the Shapiro–Wilk test, the distribution of continuous variables was evaluated. The pre- and postoperative values of each outcome parameter were compared using the paired *t* test (parametric) or the Wilcoxon test (nonparametric) for two related samples. Significance level was set at *p* = 0.05. A post hoc power analysis demonstrated a power of 99.9 percent for this study (*p* < 0.05). RStudio (Version 1.4.1717©2009-2021 RStudio, PBC, 250 Northern Ave, Suite 420, Boston, MA, USA) was used for statistics and graphics.

## 3. Results

### 3.1. Demographics

**Figure 1:**
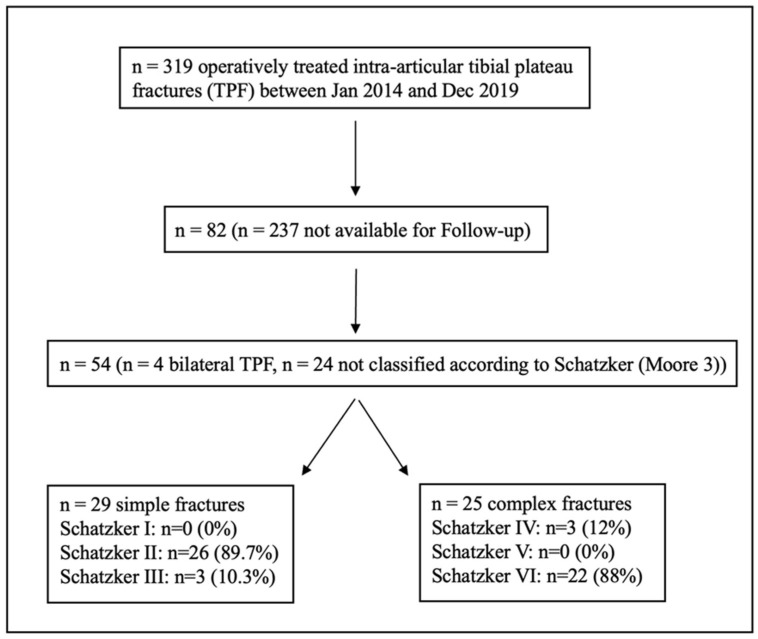
Flowchart demonstrating inclusion and exclusion criteria for exclusion.

Overall, 54 patients (mean age 51 ± 11.9 years, 59.3% female, and 40.7% male) with a mean follow-up of 3.9 years (±1.6 years; median: 3.8 years) were included; the criteria for exclusion are illustrated in Figure 1.

The epidemiological data are shown in Table 1.

### 3.2. Outcomes

The results of this study show that all scores assessing the outcome of TPFs were lower at the one-year follow-up after surgery. Significant improvements were observed between the first postoperative year and the follow-up period. The IKDC score showed good (81%) and excellent (85%) results in the simple and complex fracture groups, respectively. Similarly, the Lysholm score indicated good outcomes with 93 points for simple fractures and 90 points for complex fractures. Moreover, the VAS score improved to 0.9 points in the simple fracture group and 0.7 points in the complex fracture group at the one-year postoperative evaluation.

**Figure 2:**
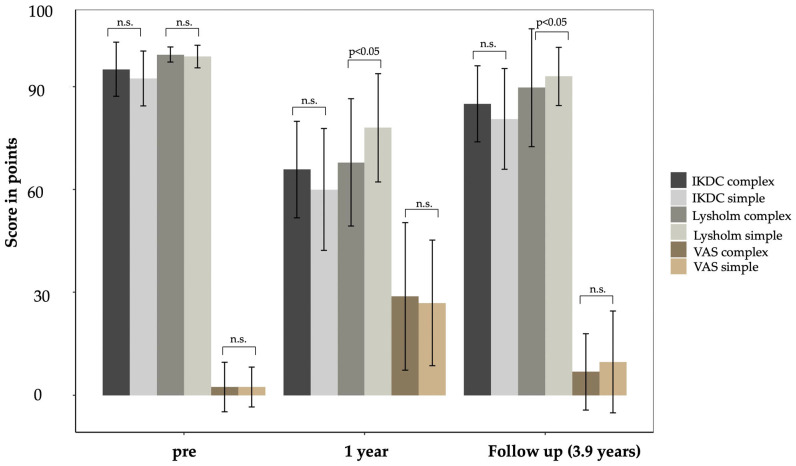
Patient-reported outcomes of both groups (complex and simple fractures) over time. The error bars in this diagram represent the mean ± standard deviation (SD), illustrating the data dispersion within each group. Furthermore, significances between the groups for each score are indicated above each column; “n.s.” stands for “not significant”.

**Figure 3:**
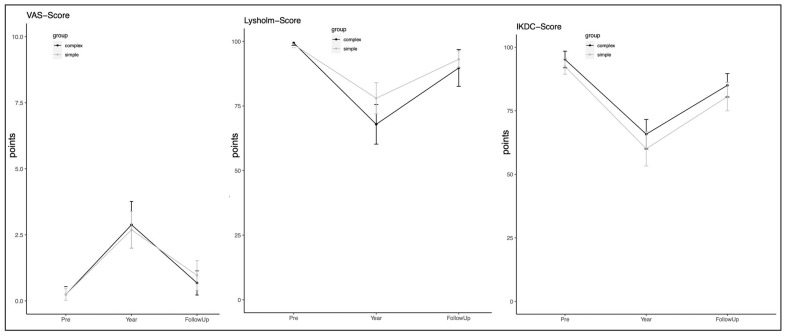
Patient-reported outcomes of both groups (complex and simple fractures) over time.

When compared between the groups, the outcomes of simple and complex fractures revealed no significant differences in the IKDC score and VAS at the follow-up assessment (Figure 2 and Figure 3). However, the Lysholm score for simple fractures was significantly higher.

Furthermore, at the one-year follow-up, patients with simple TPFs experienced a significant increase in pain (measured by VAS) and a decrease in knee function (measured by Lysholm and IKDC), compared to their pre-injury state, as presented in Table 2 and Table 3. Although the pain decreased by the 3.9-year follow-up, significant differences in pain levels persisted when compared to the pre-injury state (*p* = 0.018).

Both the IKDC and Lysholm scores showed significantly lower results at the final follow-up visit compared to the pre-injury state (*p* < 0.01). The activity level, as measured by the Tegner score, was also decreased at the one-year follow-up but had improved by the 3.9-year follow-up, with no significant differences compared to the pre-injury state (*p* = 0.357).

In complex fractures, there was a significant increase in pain levels after the first-year follow-up (*p* < 0.001), but no significant differences were observed at the final follow-up. The functional scores also showed significantly lower results after one year of surgery, except for the IKDC score. At the final follow-up, there were no significant differences in functional outcomes compared to the preinjury state.

In terms of ROM, no significant differences were found between the complex fracture group and the simple fracture group (Table 4). Both groups exhibited similar ROM in both the injured and healthy knees. However, there was a small difference in knee extension, with the complex fracture group demonstrating a slightly lower range of motion in the injured knee compared to the simple fracture group. Nonetheless, both groups achieved comparable ROM overall.

### 3.3. Return to Sports (RTS)

**Figure 4:**
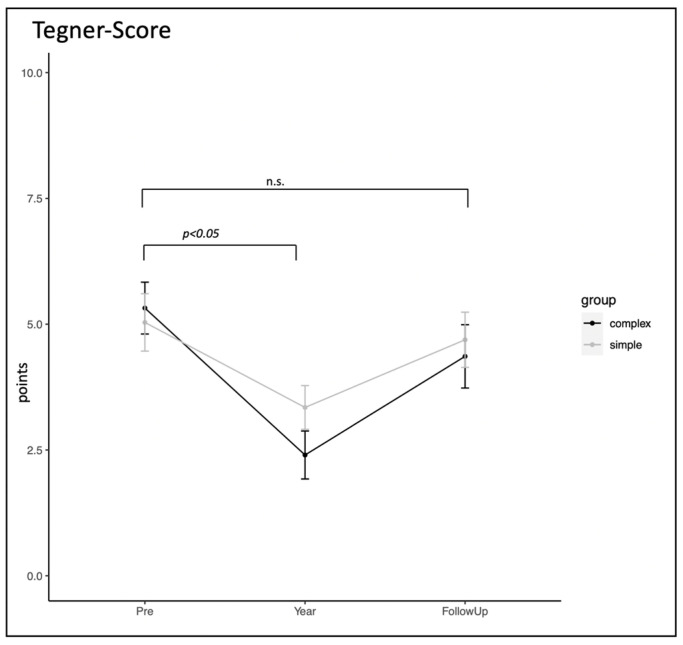
RTS measured by Tegner score; “n.s.” stands for “not significant”.

Initially, all 54 participants were active in sports. During follow-up, 100% resumed sports, with 14.8% returning to the same sport after one year. About 79.6% switched sports. After surgery, 63% went back to their original sport, and 37% chose a different one. The fracture type influenced the Tegner score: simple fractures scored higher initially (3.3 vs. 2.4), but no differences were found at follow-up (4.7 vs. 4.4). Return-to-sports rates were 100%. Adjustments were needed by 25% and 46% for simple and complex fractures. See Figure 4 for detailed results.

### 3.4. Complications

A complication was considered as such if a revision surgery was necessary. The overall complication rate was 17% (*n* = 9). Among these, 11% (*n* = 6) experienced a cartilage defect, 4% (*n* = 2) developed a post-traumatic deformity, and 2% (*n* = 1) had a loss of reduction.

## 4. Discussion

The most important finding of this study was that the results of this study indicate a significant impact of TPFs on knee function, activity level, and pain according to patient-reported outcomes. Patients experienced increased pain and decreased knee function and activity level at the one-year follow-up compared to their pre-injury state. In addition, when interpreting the data from this study, patient-reported outcomes after surgically treated TPFs are not dependent on fracture classification. Additionally, the findings suggest that patient satisfaction can be achieved in the midterm, specifically after 3 to 9 years of follow-up. It is crucial to inform patients that it may take more than a year to regain a certain level of activity, but the long-term outcomes are generally good to excellent. This underscores the importance of long-term follow-up and rehabilitation.

Even though the data from this study showed satisfactory outcomes in the short and midterm, there remains a lack of information regarding patient satisfaction over a longer timeframe of 10 to 20 years, which requires further investigation in future research. For instance, post-traumatic osteoarthritis is one of the most common long-term complications of TPFs, with rates ranging from 17% to 44% [8,26,27,28].

The results of the IKDC, Lysholm, and VAS score suggest that patients with TPFs experience a decrease in functional outcomes and an increase in pain levels at the one-year follow-up. Although improvements are observed over time, patients may still have significant differences in pain levels and functional outcomes compared to their pre-injury state. However, the range of motion (ROM) is generally comparable between simple and complex fractures, with only minor differences noted in knee extension. This finding indicates that, despite the varying severity of fractures, both groups achieved similar range of motion outcomes.

Several previous studies have consistently reported satisfactory to excellent results for TPFs, irrespective of patient age or fracture complexity [15,19,21,29]. However, complex fractures are known to be associated with numerous complications that significantly impact the functional outcome of the fracture [30]. Furthermore, this study supports the existing research showing a lack of correlation between fracture classification and outcomes, such as the Western Ontario and McMaster Universities Osteoarthritis Index (WOMAC) score or the severity of post-traumatic osteoarthritis [13,14].

It is evident that post-traumatic osteoarthritis has a multifactorial nature and involves various contributing factors, including direct and indirect cartilage damage, patient age, malalignment, articular depression, soft tissue injuries, and knee joint instability [8,26,27,28]. Therefore, when evaluating and classifying TPFs, it is crucial to consider these factors beyond the bony parameters, as they play a significant role in determining the functional outcome and the development of osteoarthritis. This study represents a significant advancement, demonstrating that patient-reported outcomes after surgically treated tibial plateau fractures (TPFs) are not dependent on fracture classification. This challenges the prevailing belief that more severe fractures result in worse outcomes. It emphasizes the need to consider TPFs not just as fractures but also as joint injuries, requiring a comprehensive treatment approach that factors in elements like knee ligament status. Notably, both simple and complex fractures yielded favorable to excellent outcomes, enabling patients to maintain an active lifestyle. However, it is important to acknowledge that a longer recovery period may be necessary, and postoperative results continue to show substantial improvement in the long run.

It is crucial to recognize that relying solely on X-ray images for predicting outcomes may not accurately reflect the actual experiences of patients. Therefore, fractures should be viewed as joint injuries, necessitating a comprehensive assessment that incorporates patient-specific factors like athleticism and the implementation of appropriate physiotherapy. Regarding the return to sports (RTS) following TPFs, while some participants had to switch sports or adjust their athletic level, the majority were able to resume their sports activities. This highlights the effectiveness of the surgical intervention and subsequent rehabilitation in facilitating an active and sports-oriented lifestyle for patients with TPFs. The high rates of RTS observed in this study challenge the common belief that these fractures may put an end to athletic careers, especially in professional sports. The study found a 100% RTS rate among participants primarily engaged in amateur sports, with a mean pre-injury Tegner score of 5.2 points. Initially, simple fractures showed higher activity levels compared to complex fractures at the one-year follow-up, but these differences equalized at the final follow-up, indicating that athletes with complex fractures have the potential to regain similar levels of activity.

However, it is crucial to inform patients about the likelihood of transitioning to lower-impact sports and adjusting their sporting level accordingly. In the literature, RTS rates of 52% [11], 73% [31], and 88% [32] have been reported, and the TPF is often referred to as a challenge for professional sports careers [12]. Nevertheless, Kugelmann et al. demonstrated that even patients who suffered polytrauma were also able to return to athletic training [11]. Complex fractures took longer to return to sports within 6 months, which aligns with our results, as, after one-year, simple fractures exhibited significantly higher activity levels than complex fractures. The authors also noted that patients had to adjust their sporting level in previous studies [11,15,31].

Another important aspect is that the Schatzker classification was first created for conventional imaging and can only partially capture the intricacy of fractures. The clinical standard of care for suspected TPFs is currently further tomographic imaging. To take into consideration the complexity of the fracture, a number of new categorization algorithms based on tomographic imaging have been developed [4,33,34]. Nevertheless, these more intricate classification schemes have poorer intra- and inter-observer reliability, and not all CT-based classifications accurately depict the fracture’s morphology at all stages. All in all, this explains why, despite the urgent need for one, none of the 38 methods detailed in the literature has yet established itself as a standard classification system [35].

Currently, there are insufficiently thorough outcome studies of both surgical and non-surgical treatment in patients of various ages. There is a lack of research from recent years that compare non-surgical treatment to surgical treatment for TPFs [4]. Only a few older studies exist emphasizing that conservative treatment is an acceptable alternative to surgery. New treatment strategies, like direct primary knee arthroplasty, are also becoming more prominent as patients age [36,37]. There is a need for investigations to determine whether surgical or non-surgical treatment offers an advantage, especially in the elder patient population.

This study has several limitations. Firstly, the sample size was relatively small, which may not fully represent the entire population of patients with TPFs. It likely focused on a specific subset of patients from a particular geographic region or healthcare setting, limiting the ability to apply the findings to a broader population. Secondly, the retrospective design of this study may lead to selection bias. Relying on retrospective data could introduce inaccuracies, incomplete records, or missing information, which may affect the overall quality of the study’s findings. Moreover, the exclusion of patients who could not be contacted, were unavailable for follow-up due to reasons such as a change in address or a language barrier (lack of proficiency in German or English), and refused participation may introduce a potential source of selection bias. Additionally, the study’s reliance on the availability of accurate contact information poses a constraint on the completeness of the dataset. Thirdly, no data on knee joint laxity were collected, an essential factor in assessing stability and functional outcomes after TPFs. The absence of these data could limit the comprehensive understanding of the patients’ recovery and treatment efficacy. Additionally, the study primarily focused on patient-reported outcomes and pain levels. While these are essential indicators of patient well-being, they may not fully capture all aspects of functional recovery or long-term treatment effects. Fourthly, the minimum one-year follow-up period might not be sufficient to capture the full extent of recovery or long-term complications that could arise from TPFs. Longer follow-up periods would provide more informative data to assess the durability of treatment outcomes. Moreover, since data on the progression of osteoarthritis was not collected, the study may not fully explore the potential long-term consequences of TPFs on joint health and function. Lastly, the study does not account for variations in treatment approaches among patients, such as surgical techniques or rehabilitation protocols, which could impact the observed outcomes.

Further research with larger and more diverse populations is needed to validate and generalize these findings, allowing for more robust conclusions. Although the study emphasizes that patient-reported outcomes following TPFs are not solely dictated by fracture severity and that patients can lead active lifestyles with low pain levels in the medium term, further research is warranted to elucidate the factors contributing to divergent outcomes among patients with TPFs. This will enable more personalized and effective treatment approaches.

## 5. Conclusions

The data from this study showed that both simple and complex tibial plateau fractures show favorable outcomes at midterm follow-up, and that injury severity does not correlate with worse results. While patients may tend to overestimate the recovery speed, this research highlights the importance of long-term follow-up, demonstrating a substantial improvement between one year post-surgery and the final evaluation. Return-to-sports rates were high, with adjustments needed for certain activities. However, patients should recognize the need to shift to lower-impact sports and the lengthy recovery process.

## Figures and Tables

**Table 1:** Demographic data of both groups (simple and complex fractures); “n.s.” stands for “not significant”.

**Criteria**	**Simple Fractures (*n* = 29)**	**Complex Fractures (*n* = 25)**	***p*-Value**
Age	49.8 ± 12.5	52.5 ± 11.4	n.s.
Female	58.6%	60%	n.s.
Male	41.4%	40%	n.s.
Schatzker			
I	*n* = 0 (0%)	*n* = 0 (0%)	-
II	*n* = 26 (89.7%)	*n* = 0 (0%)	-
III	*n* = 3 (10.3%)	*n* = 0 (0%)	-
IV	*n* = 0 (0%)	*n* = 3 (12%)	-
V	*n* = 0 (0%)	*n* = 0 (0%)	-
VI	*n* = 0 (0%)	*n* = 22 (88%)	-
Cause of accident (%)			
Fall	42.9%	23.1%	*p* < 0.05
Traffic	14.3%	26.9%	*p* < 0.05
Ski	21.4%	19.2%	n.s.
Biking	10.7%	26.9%	*p* < 0.05
Other	10.7%	3.8%	*p* < 0.05
BMI pre-injury	23.6 ± 3.6	24.8 ± 2.6	n.s.
BMI follow-up	24.7 ± 3.8	29 ± 4.5	n.s.

**Table 2:** Patient-reported outcomes of the simple fracture group; “n.s.” stands for “not significant”.

**Score**	**Pre-Injury (Simple)**	**One Year (Simple)**	**Follow-Up (Simple)**	***p*-Value**
VAS	0.24 ± 0.57	2.69 ± 1.83	0.96 ± 1.48	*p* < 0.05
Lysholm score	98.79 ± 3.29	78 ± 15.81	93.06 ± 8.49	*p* < 0.05
IKDC score	92.42 ± 8.01	60.04 ± 17.79	80.57 ± 14.70	*p* < 0.05
Tegner score	5.03 ± 1.49	3.34 ± 1.14	4.69 ± 1.44	n.s.

**Table 3:** Patient-reported outcomes of the complex fracture group; “n.s.” stands for “not significant”.

**Score**	**Pre-Injury (Complex)**	**One Year (Complex)**	**Follow-Up (Complex)**	***p*-Value**
VAS	0.24 ± 0.72	2.88 ± 2.15	0.68 ± 1.10	n.s.
Lysholm score	99.40 ± 2.19	67.92 ± 18.61	89.72 ± 17.25	n.s.
IKDC score	95.17 ± 7.91	65.79 ± 14.12	85.01 ± 11.15	n.s.
Tegner score	5.32 ± 1.25	2.4 ± 1.15	4.36 ± 1.52	n.s.

**Table 4:** Range of motion of the injured and healthy leg between complex and simple fractures; “n.s.” stands for “not significant”.

**Range of Motion**	**Complex Fractures**	**Simple Fractures**	***p*-Value**
Flexion injured	126 ± 14	127 ± 7	n.s.
Flexion healthy (control)	129 ± 6	129 ± 0	n.s.
Extension injured	2 ± 1	1 ± 1	n.s.
Extension healthy (control)	2 ± 0	2 ± 0	n.s.

## Data Availability

The original contributions presented in the study are included in the article; further inquiries can be directed to the corresponding author.
